# Effects of Glucose Intolerance on Physiological Accumulation in Salivary Glands and Palatine Tonsils During 18F-Fluorodeoxyglucose Positron Emission Tomography

**DOI:** 10.7759/cureus.67387

**Published:** 2024-08-21

**Authors:** Shoki Nakamura, Koya Nakatani, Kumiko Yoshino, Takashi Koyama

**Affiliations:** 1 Department of Diagnostic Radiology, Kurashiki Central Hospital, Kurashiki, JPN; 2 Department of Diagnostic Imaging and Nuclear Medicine, Kyoto University Hospital, Kyoto, JPN; 3 Department of Diagnostic Imaging and Nuclear Medicine, Kyoto University Graduate School of Medicine, Kyoto, JPN

**Keywords:** physiological accumulation, hyperglycemia, fdg-pet, oral cavity, tonsil, salivary gland

## Abstract

Purpose: We evaluated the effects of chronic hyperglycemia on physiological accumulation in salivary glands and tonsils during ^18^F-fluorodeoxyglucose positron emission tomography (FDG-PET/CT).

Materials and methods: 12,738 patients underwent whole-body FDG-PET/CT in our institute during the study period. Of these, the case group comprised 777 patients with a blood glucose (BG) level >140 mg/dL; the control group comprised an equal number of randomly selected age- and sex-matched individuals with a BG level <110 mg/dL. Within the case group, the diabetic subgroup was defined as individuals with a BG level >200 mg/dL. Visual assessment and accumulation intensity among tissues were compared between the case and control groups, including (1) the mean difference in maximum standardized uptake value (SUVmax), (2) the difference in the proportion of patients with visible tissues on maximum intensity projection images, and (3) differences between the diabetic subgroup and the control group.

Results: Parotid, submandibular, sublingual, and tonsillar tissues all showed significantly lower SUVmax in the case group than in the control group. The proportions of individuals with visible uptake in the parotid and tonsillar tissues and in the sublingual gland were significantly smaller in the case group than in the control group. Tonsillar uptake was observed in more than 90% of individuals in the control group but in two-thirds of patients in the diabetic subgroup. Accumulation in the parotid and submandibular glands was visible in approximately 80% of individuals in the control group but only half of patients in the diabetic subgroup.

Conclusion: Physiological accumulation in salivary glands and tonsils is significantly reduced among individuals with hyperglycemia or diabetes.

## Introduction

Positron emission tomography (PET) using 18F-fluorodeoxyglucose (FDG) is one of the most useful whole-body imaging modalities for cancer. An understanding of physiological accumulation is important when evaluating and interpreting FDG-PET images, but the distribution varies among individuals and many aspects require clarification. FDG accumulation depends on various factors that increase glucose uptake, such as molecular shifts of glucose transporters in cells and phosphorylation/dephosphorylation of sugar chains [1-3]. A recent systematic review of the relationship between blood glucose and FDG accumulation was published and concluded that increased blood glucose significantly correlated with decreased FDG accumulation in the brain and increased FDG accumulation in the liver and blood pool [4,5]. In addition, in tumors, FDG accumulation was decreased in the hyperglycemia group with blood glucose levels exceeding 200 mg/dl, while no significant correlation was found between lower blood glucose levels and FDG accumulation [5]. These are important findings for interpreting FDG-PET images. However, the relationship between physiological accumulation and blood glucose levels in various organs remains unclear. In particular, physiological accumulation in the salivary glands, tonsils, and oral cavity remains largely uncharacterized in terms of its mechanism, as well as the phenomena it reflects and its pathological significance. Typical distribution patterns of physiological accumulation in the head and neck region have been reported [6]. A proposed criterion for classifying accumulation in salivary gland and tonsillar tissue as physiological or pathological involves determining whether there is a >1.5-fold difference in accumulation intensity between the left and right sides [7]. However, there is considerable variation in physiological accumulation intensity; because the mechanism has not been characterized, the use of a cutoff for convenience leads to unnecessary scrutiny and is not always appropriate in clinical practice.

In our daily clinical practice, we often experience that the physiological accumulation in the salivary glands and tonsils is markedly reduced compared to healthy subjects in cases of suspected chronic hyperglycemia with blood glucose levels exceeding 200 mg/dL at the time of examination. Although it can be challenging to elucidate some features of physiological accumulation among oral tissues, we have noticed in our clinic that strong physiological accumulation in submandibular glands is often accompanied by physiological accumulation in tonsils. It has been shown that macrophages and granulation tissue contribute significantly to FDG accumulation in addition to tumor tissue, and FDG accumulation on FDG-PET images reflects not only tumor cells but also nonneoplastic changes caused by macrophages and other cells [8]. We hypothesize that oral immunity is involved in these physiological accumulation phenomena. Diabetic patients have decreased salivary secretory capacity and compromised immune status [9]. If salivary gland and tonsillar physiological accumulation are decreased in patients with chronic hyperglycemia or diabetes compared with healthy controls, our hypothesis could be supported. An exploration of the factors involved in salivary gland and tonsillar physiological accumulation may enable a more accurate interpretation of the pathological significance.

This study explored the effects of hyperglycemia and diabetes on physiological accumulation in the oral cavity through epidemiological investigation of the characteristics of salivary gland and tonsillar physiological accumulation on FDG-PET/computed tomography (CT) images of patients with chronic hyperglycemia; it also examined correlations between tissues. This knowledge will help to avoid common errors during the FDG-PET evaluation of patients with head and neck cancer or hematological diseases, while also providing insights concerning the mechanisms underlying the physiological accumulation of FDG.

## Materials and methods

Participants

This case-control study enrolled all individuals who underwent whole-body FDG-PET/CT between 2016 and 2021 in our hospital. Patients were excluded if the scan was not covered by insurance (including health screening) if the scan was performed for the assessment of head and neck cancer or hematological disease, if they had clinically documented lesions in the salivary glands or tonsils, or if they declined to participate in this study. The case group comprised patients with a blood glucose (BG) level >140 mg/dL. The control group comprised an equal number of age- and sex-matched individuals with a BG level <110 mg/dL and no history of chronic hyperglycemia or diabetes; these individuals were selected from the Radiological Information System (RIS) database using a random number table. Within the case group, the diabetic subgroup was defined as individuals with a BG level >200 mg/dL. This retrospective study protocol was approved by the local medical ethics committee. The procedures in this study were conducted in accordance with ethical standards established in the 1964 Declaration of Helsinki. Because the study used only anonymized clinical data, consent was obtained in an opt-out format; rather than providing a written and oral explanation to each patient, an overview of the study was publicly displayed, and patients were provided with a clear opportunity to opt out of the study.

FDG-PET/CT scanning protocol

All patients were asked to fast for at least four hours before FDG administration. Blood glucose levels were measured at the time of the FDG injection. In this study, no examinations were postponed because of hyperglycemia. The FDG activity administered to each patient was 185 MBq on the calibration date (18F-FDG scan injectable; Nihon Medi-Physics, Tokyo, Japan). After FDG administration, the patient was taken to a resting area. PET/CT images were acquired approximately one hour after FDG administration. All scans were performed using a Biograph mCT PET/CT scanner (Siemens Healthineers, Erlangen, Germany). Emission scans were sequentially performed after CT scans with an acquisition time of two minutes per bed position, and images were reconstructed using a three-dimensional (3D) iterative reconstruction algorithm: 3D-OSEM+PSF+TOF.

Image interpretation and data analyses

First, we evaluated whether accumulation in the parotid gland, submandibular gland, sublingual gland, and tonsillar tissue could be visually recognized on whole-body maximum intensity projection (MIP) images of the case and control groups. Second, accumulation intensity was determined by measuring the maximum standardized uptake value (SUVmax) of each tissue in each group. Visual assessment and accumulation intensity were investigated similarly among individuals in the diabetic subgroup.

Visual assessment and accumulation intensity among tissues were compared between the case and control groups, including (1) the mean difference in SUVmax, (2) the difference in the proportion of patients with visible tissues on MIP images, and (3) differences between the diabetic subgroup and the control group. As a supplemental analysis, correlations of SUVmax between salivary glands and tonsils were also assessed.

Statistical analyses were performed using GraphPad Prism (Dotmatics, Boston, MA, USA). Unpaired t-tests were used to compare the mean SUVmax data. Fisher's exact test was used to compare the proportions of patients with visible tissues. In all statistical tests, the significance threshold was defined as p<0.05. Correlations of SUVmax between salivary glands and tonsils were determined by Pearson correlation coefficients.

## Results

In total, 12,738 patients underwent whole-body FDG-PET/CT in our institute during the study period. Of these, 777 patients (505 men and 272 women) were enrolled as the case group; their mean age was 71.4 (range 22-91) years, and their mean BG level was 172.1 (range 142-440) mg/dL. Within the case group, 126 patients (91 men and 35 women) were classified as the diabetic subgroup; their mean age was 70.8 (range 40-89) years, and their mean BG level was 244.0 (range 200-440) mg/dL. For the control group, 777 patients (505 men and 272 women) were identified by stratified random sampling; their mean age was 71.5 (range 23-93) years, and their mean BG level was 97.0 (range 61-109) mg/dL (Table 1).

**Table 1:** Group profiles F: female; M: male

	Case group	Diabetic subgroup	Control group
No. of patients	777 (F/M=272/505)	126 (F/M=35/91)	777 (F/M=272/505)
Age, years (mean ± SD [range])	71.4 ± 9.1 [22–91]	70.8 ± 9.5 [40–89]	71.5 ± 9.4 [23–93]
Blood glucose, mg/dL (mean ± SD [range])	172.1 ± 38.8 [142–449]	244.0 ± 44.0 [200–449]	97.2 ± 8.7 [61–109]

**Figure 1:**
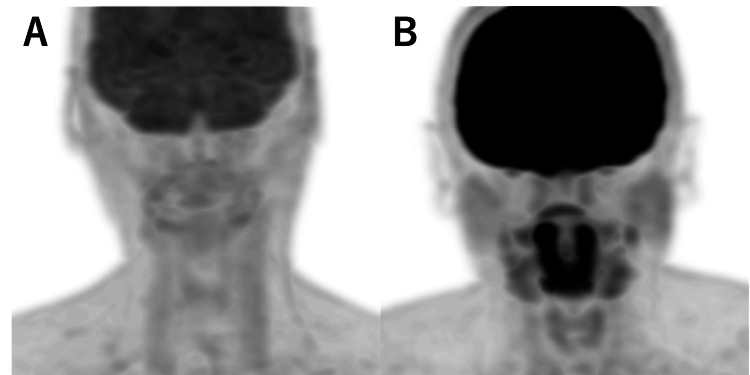
Representative images of the control and case groups A: A typical case for the case group; 80’s male with gastric cancer; blood glucose 204 mg/dL, SUVmax: parotid 1.2 submandibular 1.6 sublingual 1.6 palatine tonsils 1.7. B: A representative case of the control group; 50’s male with lung cancer; blood glucose 83 mg/dL, SUVmax: parotid 2.1 submandibular 3.2 sublingual 5.2 palatine tonsils 7.7.

**Figure 2:**
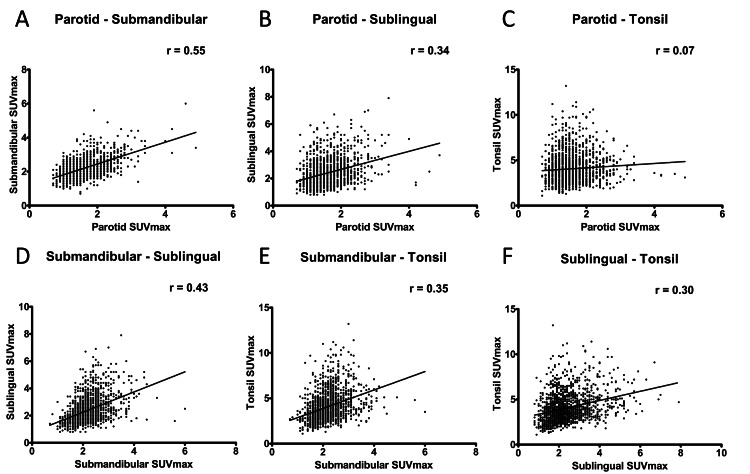
Correlations of SUVmax among salivary glands and tonsils across case and control groups (both groups were included)

Representative images of each case group and control group are shown (Figure 1). The mean SUVmax of the parotid gland, submandibular gland, sublingual gland, and tonsils were significantly lower in the case group than in the control group (Table 2). Moreover, the diabetic subgroup showed lower SUVmax in all salivary glands. Regarding the tonsils, individuals in the case and control groups had SUVmax >10, but this result was not present among individuals in the diabetic subgroup. The parotid glands and tonsils were visible on 77% and 91% of MIP images in the control group, respectively; they were visible on fewer MIP images in the case group (49% and 81%, respectively; both p<0.0001). Sublingual glands were visible on 63% of MIP images in the control group and 58% of MIP images in the case group (p=0.017). Submandibular glands were visible in 81% of MIP images in the control group and 80% of MIP images in the case group; this difference was not statistically significant (p=0.32). However, when restricted to the diabetic subgroup, the proportion of visible submandibular glands was 55%, which was significantly lower than the proportion in the control group (p<0.0001). Visible proportions in the diabetic subgroup were 38% for sublingual glands, 65% for tonsils, and 46% for parotid glands; these were all significantly lower than the proportions in the control group (all p<0.0001) (Table 3). Supplemental analyses revealed a moderately strong correlation between SUVmax in the parotid gland and SUVmax in the submandibular gland (r=0.55). SUVmax in the tonsils exhibited a weak correlation with SUVmax in the submandibular gland (r=0.35) and a minimal correlation with SUVmax in the parotid gland (r=0.07) (Figure 2).

**Table 2:** Differences in SUVmax among salivary glands and tonsils *p<0.05 was used to define statistical significance. SD: standard deviation.

	Case group Mean ± SD [range]	Diabetic subgroup Mean ± SD [range]	Control group Mean ± SD [range]	p-value Case vs. control	p-value Diabetic vs. control
Parotid gland	1.4 ± 0.4 [0.7–3.0]	1.3 ± 0.3 [0.7–2.6]	1.7 ± 0.5 [0.7–4.9]	p<0.0001*	p<0.0001*
Submandibular gland	2.0 ± 0.4 [0.7–3.8]	1.8 ± 0.3 [1.2–2.9]	2.4 ± 0.6 [1.0–6.0]	p<0.0001*	p<0.0001*
Sublingual gland	2.2 ± 0.8 [0.8–6.1]	1.8 ± 0.5 [0.9–4.2]	2.6 ± 1.1 [0.8–7.9]	p<0.0001*	p<0.0001*
Tonsil	3.6 ± 1.3 [1.1–10.6]	2.8 ± 0.9 [1.1–5.6]	4.5 ± 1.7 [1.4–13.2]	p<0.0001*	p<0.0001*

**Table 3:** Differences in proportions of visually discernible cases among groups *p<0.05 was used to define statistical significance.

	Case group (n=777)	Diabetic subgroup (n=126)	Control group (n=777)	p-value Case vs. control	p-value Diabetic vs. control
Parotid gland	384 (49%)	58 (46%)	595 (77%)	<0.0001*	<0.001*
Submandibular gland	624 (80%)	69 (55%)	632 (81%)	0.320	<0.001*
Sublingual gland	448 (58%)	48 (38%)	490 (63%)	0.017*	<0.001*
Tonsil	633 (81%)	82 (65%)	709 (91%)	<0.001*	<0.001*

## Discussion

In this study, the parotid, submandibular, sublingual, and tonsillar tissues all showed significantly lower SUVmax in the case group than in the control group. In tonsillar tissues, there were some instances of very strong accumulation, including an individual in the control group with SUVmax >10; there were no such findings in the diabetic group. The proportions of individuals with visible uptake in the parotid and tonsillar tissues and in the sublingual gland were significantly smaller in the case group than in the control group; no significant difference was observed regarding the submandibular gland. However, when the comparison was limited to the diabetic group, the proportion of individuals with visible uptake in submandibular glands was significantly smaller; this trend also extended to the other salivary glands. Tonsillar uptake was observed in more than 90% of individuals in the control group but in two-thirds of patients in the diabetic subgroup. Accumulation in the parotid and submandibular glands was visible in approximately 80% of individuals in the control group, but only half of patients in the diabetic subgroup. Correlations of accumulation intensity among organs, assessed in supplemental analyses, were slightly stronger between parotid and submandibular glands. Tonsillar accumulation exhibited a weak correlation with submandibular gland accumulation and a minimal correlation with parotid gland accumulation.

These results were consistent with our initial hypothesis that physiological uptake in the salivary glands and tonsils would be lower in patients with diabetes. However, considering that significant differences were identified at all sites, it remains possible that these differences simply reflect a decrease in the cell membrane density of glucose transporter (Glut)-1 among various organs as the BG level increased. Because Glut-1 is considered primarily responsible for glucose uptake in the brain [10-12], further examination of its relationship with physiological brain accumulation may provide relevant insights. The substantial reduction in the frequency of visible tonsillar physiological accumulation in patients with diabetes suggests that diabetes can affect tonsillar function. We speculate that this effect is related to the reduced oral immune function observed in diabetic patients [13-15].

The reduced FDG accumulation in salivary glands and tonsils shown in this study in patients with diabetes may be explained by abnormalities of oral immunity in patients with diabetes. In order to identify pathological FDG accumulation on FDG-PET imaging, the meaning of physiological accumulation in each organ must be correctly understood. FDG is known to accumulate not only in tumors but also in inflammatory changes, often causing false-positive and false-negative results in FDG-PET imaging [16,17]. In vivo, experiments have shown that FDG also accumulates in macrophages and granulation tissue and that GLUT-1 and GLUT-3, which are involved in FDG accumulation, are also expressed in inflammatory cells [8,18]. Although it has been shown that macrophages may play an important role in FDG accumulation, the role of macrophages in salivary glands and tonsils is unclear [19,20]. Further basic research is needed on how resident macrophages in the salivary glands and tonsils are involved in the organized accumulation of FDG.

A key limitation of this study was the occasional difficulty distinguishing between physiological accumulation in sublingual glands and physiological accumulation in skeletal muscles such as the proximal external hyoid muscles (e.g., mandibular bicuspid muscle). Additionally, the study population included small numbers of underweight and overweight patients, which may have influenced the weight-normalized SUV. The use of a maximum or peak standardized uptake value corrected for lean body mass (SULmax or SULpeak, respectively) as an alternative metric is a potential solution to this limitation [21,22]. In future research, it would be insightful to investigate the relationship between obesity and physiological accumulation in these organs. The present study focused on FDG-PET findings and pre-test blood glucose levels, but future studies are needed to provide histopathological support for FDG accumulation in the salivary glands and tonsils. In addition, the phenomenon may have more meaningful clinical implications by limiting the target patient population and elucidating its relationship to disease diagnosis, patient prognosis, and survival.

## Conclusions

In both salivary gland and tonsillar tissues, the case group had significantly lower physiological accumulation than the control group; moreover, the frequency of visible accumulation was significantly lower in the diabetes subgroup.

The significant differences in physiological uptake observed in all salivary and tonsillar glands may reflect a decrease in the cell membrane density of Glut-1 in each organ with increasing BG levels. New insights may be gained by examining the relationship between physiological brain accumulation and Glut-1 expression in the brain. The decreased accumulation in the tonsils of diabetic patients may reflect the oral immune dysfunction known to occur in diabetic patients. Further basic research is needed on the physiological function of the tonsils, their relationship to immune cells, and the differences between healthy individuals and diabetic patients.
